# The evolution of character education in medicine

**DOI:** 10.1080/10872981.2025.2607813

**Published:** 2025-12-26

**Authors:** Julie K. Silver, Amber Brooks, Suzanne C. Danhauer, Chris Gillette, Roshell Muir, Darcy Reed, Mary R. Shen, Anna R. Silver, Ethan Stonerook, Kenneth Townsend

**Affiliations:** a Department of Physical Medicine and Rehabilitation, Wake Forest University School of Medicine, Winston-Salem, NC, USA; b Department of Anesthesiology, Wake Forest University School of Medicine, Winston-Salem, NC, USA; c Department of Social Sciences & Health Policy, Wake Forest University School of Medicine, Winston-Salem, NC, USA; d Department of PA Studies, Department of Epidemiology and Prevention, Wake Forest University School of Medicine, Winston-Salem, NC, USA; e Faculty Development, Office of Faculty Affairs, Wake Forest University School of Medicine, Winston-Salem, NC, USA; f Department of Internal Medicine, Wake Forest University School of Medicine, Winston-Salem, NC, USA; g Department of Psychiatry, Harvard Medical School, Boston, MA, USA; h Center for Anxiety and Related Disorders, Boston University, Boston, MA, USA; i Department of PA Studies, Wake Forest University School of Medicine, Winston-Salem, NC, USA; j Leadership and Character in the Professional Schools, Wake Forest University, Winston-Salem, NC, USA

## Introduction

The evolution of character education represents an important development in medical education and leadership training. As character education becomes a more important part of training, it must translate across domains (e.g., philosophy, psychology, technology); disciplines (e.g., research, clinical care); and specialties (e.g., obstetrics and gynaecologist, psychiatry, neurosurgery). Although in this article we focus primarily on physicians and physicians-in-training, character education needs also to support interprofessional collaboration (e.g., nurses, advanced practice providers, social workers). Character education should embrace tenets of medical education: practicing the principle of beneficence, valuing evidence-based medicine, focusing on patient-centred care, embracing inclusive excellence, fostering a sense of belonging, and supporting the well-being of all.

## A historical perspective

Writing around 350 B.C., Aristotle is widely credited with providing the first holistic description of the importance of a person's character [1]. This early description helped create the branch of moral philosophy that emphasises an individual's character and virtues in assessing ethical behaviour. Moral philosophy posits three general theories of ethics, which focus on character development (virtue ethics); rules (deontology); and consequences (consequentialism). Since Aristotle's initial description, the study of character has evolved. Given this history, it is no surprise that the literature is vast, complex, and filled with intellectual disagreement. For example, some advocate for an evolved ‘neo-Aristotelian’ approach while others believe that a pro-social psychology-based approach is preferable [2]. Fortunately, there is consensus on the need for people to behave in an ethical manner.

In medicine, as in other fields, character education is primarily concerned with supporting people in developing moral and ethical behaviour that is aligned with societal expectations as well as professional expectations. Character virtues and traits have been characterised in various ways. For example, Peterson and Seligman developed the Values in Action (VIA) project that both classified character virtues and traits as well as evaluated them using various tools and participant age groups [3].

In considering the field of character development, one aspect that has, paradoxically, both helped and hindered the field of character development is how most of the world's major religions ascribe to virtue ethics. Undeniably, the religious adoption of virtue ethics has led to wide propagation. For some, this alignment has enhanced their own endorsement, and for others, this association has led to rejection and mistrust. Historically, there are many examples of how religion and public health intersect leading to a similar standoff between endorsers and rejectors. For example, in her book, *Ultimate Fitness: The Quest for Truth About Health and Exercise*, science journalist Gina Kolata described how in the middle of the nineteenth century, American doctors were deeply concerned with decreasing rates of physical activity as more people moved into cities [4]. Kolata explains that exercise was promoted as being good for the spirit and character (i.e., a moral obligation). So-called ‘muscular Christianity’ gained prominence at the end of the 19th century, for instance, as reflected in the rise of bicycling, an activity that promised physical as well as spiritual growth. Today, health professionals neither encourage nor discourage exercise based on any historical association with religion. Instead, they endorse physical activity based on its innate health benefits. Indeed, many religions adopt various practices that support the health and well-being of their followers.

Since politics and religion often overlap, it is not surprising that, like the religious examples above, political affinities have both helped and hindered the adoption of virtue ethics [5]. On the one hand, the idea that virtues are inherently positive and promote pro-social behaviour gives credence to character education. In contrast, political extremists have used virtue ethics and character education in ways that promote civil unrest. While reflecting upon character's association with religion, spirituality, and politics is reasonable, we suggest that character education should not be endorsed or set aside simply because of these intersections but rather evaluated on its own merit.

## Modern character education

Character education promotes both positive motivations and the skills necessary to live out those motivations. Virtues are ways of thinking, feeling, and acting that assist individuals to behave in adherence with their values. Being able to embrace one's values is central to flourishing which has been defined as ‘a person's sense of wholeness, that can be measured across domains of happiness, life satisfaction, physical and mental health, meaning and relationships’ [6].

Character-based approaches to professionalism and professional identity formation can also facilitate leadership development among learners. Medical professionals are expected to exert leadership, i.e., to steward relationships of influence toward a shared purpose, but medical training does not inevitably provide such training. Emerging scholarship highlights the degree to which various strategies, such as engaging moral exemplars and practicing self-reflection, can be used to develop effective and virtuous leadership.^1^

Interestingly, character and professionalism education are similar constructs (Table 1). As defined by Cruess and Cruess, professionalism is the expression of behaviours that are consistent with the values of the profession that are outwardly expressed to facilitate assessment [7]. Many medical educators are complementing learners' professionalism with education about professional identity formation (PIF). PIF is defined as the merging of medical learners' self- and professional identities so that they see themselves as possessing the inherent characteristics of being a physician [7]. Similarly, character education has overlap with psychological ‘pro-social behaviour’ which are voluntary behaviours that benefit other individuals or society in general [8]. For example, these include behaviours that demonstrate cooperation, trustworthiness, kindness, fairness, and generosity.

**Table 1.** Comparison of character and professionalism.

	Character	Professionalism
Key quality	Specific virtues and character traits are believed to direct an individual's actions, for example empathy, integrity, humility, and wisdom.	Outward expression of values within professional boundaries.
Behaviour	Centreing on virtue ethics and an individual's positive moral values.	Emphasises consistent demonstration of defined behavioural standards and accepted code of conduct. Professionalism can vary depending on the industry, organisation, and even department.
Impact	Contributes to building long-term relationships and respectability.	Focuses on establishing guidelines to direct desired individual and collective behaviours within an organisation or workplace.
Purpose	Enhances the connection between an individual's core purpose in life and commitment to their work.	Prioritises ongoing development toward high standards of competence and excellence.

The constructs of professional identity formation (PIF) and character formation (CF) have increasingly complemented professionalism in curricula. PIF and CF are rooted in positive psychology and virtue ethics, respectively, and theorise that behaviours and actions are the inevitable fruit of values and ethics internalised at the level of a person's vocational and self- identities [9,10]. The learner not only ‘knows’ or ‘does’ professionally, but ‘is’ a professional consistently, from their deeply held values and beliefs to their attitudes, behaviours, and communication with others.^2^ One challenge with this move from outward observable behaviour to internalised identity is in the ability to measure and assess these internalised phenomena [11].

Prior to the wide-spread adoption of competency-based education in medicine, character assessments were typically made and communicated through two general means. The first of these is the personal letter of recommendation, written by a trainee's supervisor or mentor for promotion to training or to independent practice. These partial accounts, however, fail to provide a basis for a comprehensive assessment of character [12,13]. The second method for assessing character historically has been through the construct of ‘professionalism’, which focuses on the outward expression of behaviours that are acceptable to other members of the profession. As Cruess and Cruess argue, this method fails to accomplish the highest ideal of medical training: producing clinicians who internally embody the profession's values, also known as professional identity [11].

There have been few interventions focused on character education in health professionals [14]. Even fewer studies have produced meaningful evaluations of the effectiveness of this education. This clear gap in the medical education literature necessitates rigorously designed methods and assessments to isolate the effectiveness of character training. There is an emerging literature that highlights the importance of virtue ethics, care ethics, and character education in medical education. For example, one report that focuses on critical questions to ask about medical education to foster flourishing suggests that fundamental values of medical education should include relevant character topics such as curiosity, empathy, integrity, humility, teamwork, and service [15]. Another review suggests that rather than using the common term burnout, it may be more appropriate to describe the challenges of medical education more as a ‘deficiency of the virtue of hope’ [16]. Further, while virtue ethics (and the idea of professionalism) focus on the individual behaviour and character of the physician (or other healthcare professional), care ethics draws in the critical importance of interdependence and trustworthiness [17] which is likely to enhance communities of practice that, in turn, help to foster individual well-being and flourishing [18] in addition to a collective sense of well-being. Such character concepts are important in education as well as the future practice of medicine, and the literature has shown greater attention to these topics in recent years. Cianciolo and colleagues argued that a systems-level approach to character training in medical education is needed. Specifically, they argue for a moral community to develop physicians using a holistic approach to create a ‘whole person’ who will then be a ‘good doctor [19].’ To date, much of the work has focused on whether character education is beneficial for living a good/whole life and not necessarily on whether patient outcomes are improved, which are both synergistically feasible and important in promoting character education [19–21].

Notably, although our central focus is on the development of a set of core virtues among all physicians, different specialties may call on different aspects of theoretical perspectives in practice. For instance, in specialties with longitudinal, relational contact with patients (i.e., psychiatry and primary care), character education may be informed by relational virtues including empathy and attunement. In procedural and acute-care fields (i.e., emergency medicine, surgery), providers may benefit from teaching of virtue-ethics constructs (i.e., stoic decision-making, practical wisdom), and in laboratory-based specialties (i.e, pathology, radiology) may require development of epistemological virtues (i.e., rigour, accuracy, and precision). Thus, when considering character education, a unifying approach must be grounded on a strong foundation of shared character values while considering the nuances that each specialty requires.

## Why character and professionalism matter in medicine

Character and professionalism incorporate qualities that are intertwined to embody learners' and physicians' individual and collective commitment to medicine [20,22]. Healthcare is generally a deeply trusted profession predicated on high moral standards [18], and both character and professionalism are required for development of successful relationships with learners, colleagues, and for maintaining ethical and respectful conduct with patients [23,24]. While both are also essential for individual and collective success, there is evidence in support of their importance in maintaining a positive work environment, healthy organisational culture and improved performance, greater job satisfaction, and better healthcare outcomes [25].

## Conclusion

Character education and leadership training complements existing practices including professionalism and PIF. There is a need to expand the literature and more rigorously study interventions. Practical wisdom dictates that academic medicine and beyond should consider incorporating character-based education during training and continuing throughout physicians' careers.

## Endnotes

1 Lamb M, Brant J, Brooks E. How is virtue cultivated? Seven strategies for postgraduate character development. *J Character Educ*. 2021;17(1).

2 Vignoles VL, Schwartz SJ, Luyckx K. Introduction: Toward an integrative view of identity. In: Schwartz S, Luyckx K, Vignoles V, eds. *Handbook of Identity Theory and Research*. Springer; 2011. doi: 10.1007/978-1-4419-7988-9_1
